# Lack of PTEN in osteocytes increases circulating phosphate concentrations by decreasing intact fibroblast growth factor 23 levels

**DOI:** 10.1038/s41598-020-78692-6

**Published:** 2020-12-09

**Authors:** Masanobu Kawai, Saori Kinoshita, Keiichi Ozono, Toshimi Michigami

**Affiliations:** 1 Department of Bone and Mineral Research, Research Institute, Osaka Women’s and Children’s Hospital, 840 Murodo-cho, Izumi, Osaka 594-1101 Japan; 2 Department of Pediatrics, Osaka University Graduate School of Medicine, Suita, Osaka Japan

**Keywords:** Physiology, Bone

## Abstract

Fibroblast growth factor 23 (FGF23) has been centric to the regulation of phosphate (Pi) metabolism; however, the regulatory network of FGF23 in osteocytes has not yet been defined in detail. We herein investigated the role of PTEN (phosphatase and tensin homolog deleted from chromosome 10) in this regulation. We created mice lacking PTEN expression mainly in osteocytes by crossing *Pten*-flox mice with *Dmp1*-Cre mice. The lack of PTEN in the osteocytes of these mice was associated with decreased skeletal and serum intact FGF23 levels, which, in turn, resulted in reductions of urinary Pi excretion and elevations of serum Pi levels. Mechanistically, the knockdown of PTEN expression in osteoblastic UMR106 cells activated the AKT/mTORC1 (mechanistic target of rapamycin complex 1) pathway and this was associated with reductions in *Fgf23* expression. Furthermore, the suppression of *Fgf23* expression by PTEN knockdown or insulin simulation in UMR106 cells was partially restored by the treatment with the mTORC1 inhibitor, rapamycin. These results suggest that FGF23 expression in osteoblastic cells is in part regulated through the AKT/mTORC1 pathway and provide new insights into our understanding of the regulatory network of Pi metabolism.

## Introduction

Phosphate (Pi) plays pleiotropic roles in a wide range of biological processes, including bone metabolism, cell signaling, structural integration, and energy homeostasis^1–4^, and fibroblast growth factor 23 (FGF23) is centric to this regulation^4–9^. FGF23 is an endocrine factor mainly produced by osteocytes that canonically acts on tissues expressing the FGF receptor and α-Klotho, such as the kidney, to regulate Pi homeostasis; the activation of the FGF23 signaling pathway decreases serum Pi levels by enhancing urinary Pi excretion. This is accomplished by reducing the localization of type IIa and IIc sodium-phosphate (Na^+^/Pi) co-transporters at the brush border membrane (BBM) of the proximal tubules in the kidney. In addition, the activation of the FGF23 signaling pathway reduces serum 1,25-dihydroxy vitamin D (1,25(OH)_2_D) levels by increasing and decreasing the expression levels of *Cyp24a1* and *Cyp27b1*, respectively.

The regulation of FGF23 in osteoblast-lineage cells, including osteocytes, has been extensively examined, and the activation of vitamin D receptor (VDR) signaling has been shown to increase the expression of *FGF23* through the VDR response element located in the promoter region of the *FGF23* gene^10,11^. Furthermore, parathyroid hormone^12–14^, the activation of the FGF receptor (FGFR) pathway, hypoxia-inducible factor 1 (HIF1) activation, sympathetic activation^12^, and inflammatory signaling^15,16^ have been implicated in the regulation of *FGF23* expression in bone; however, the transcriptional regulation of *FGF23* in osteoblastic cells has not yet been defined in detail. Accumulating evidence from clinical and animal research recently revealed a new regulatory mechanism of *FGF23* expression in bone that includes the activation of the insulin signaling pathway, with the activation of AKT reducing FGF23 expression^17^. In clinical settings, an inverse relationship was found between serum FGF23 levels and fasting insulin levels^17,18^, which supports insulin signaling being involved in FGF23 regulation in bone. However, the mechanisms by which the insulin/AKT signaling pathway affects the regulation of FGF23 expression have not yet been elucidated. In addition, in vivo evidence to support this scenario is limited.

In the present study, we introduced a model in which AKT signaling was activated by deleting PTEN (Phosphatase and Tensin Homolog Deleted from Chromosome 10). PTEN is a molecule that is known to antagonize insulin-induced AKT activation and its loss of function has been shown to activate mechanistic target of rapamycin (mTOR) complex 1 (mTORC1) pathway and block forkhead box protein O1 (FOXO1) activity^19^. By using this model, we have provided evidence to show that the lack of PTEN in osteocytes in vivo increased serum Pi levels by decreasing intact FGF23 levels. In addition, knock-down of PTEN in osteoblastic UMR106 cells in vitro reduced *Fgf23* expression and this was in part associated with the activation of the AKT/mTORC1 pathway. These results highlight the important roles of PTEN/AKT signaling in the regulation of FGF23 expression and Pi metabolism.

## Results

### Generation of osteocyte-specific *Pten*-deficient mice

**Figure 1:**
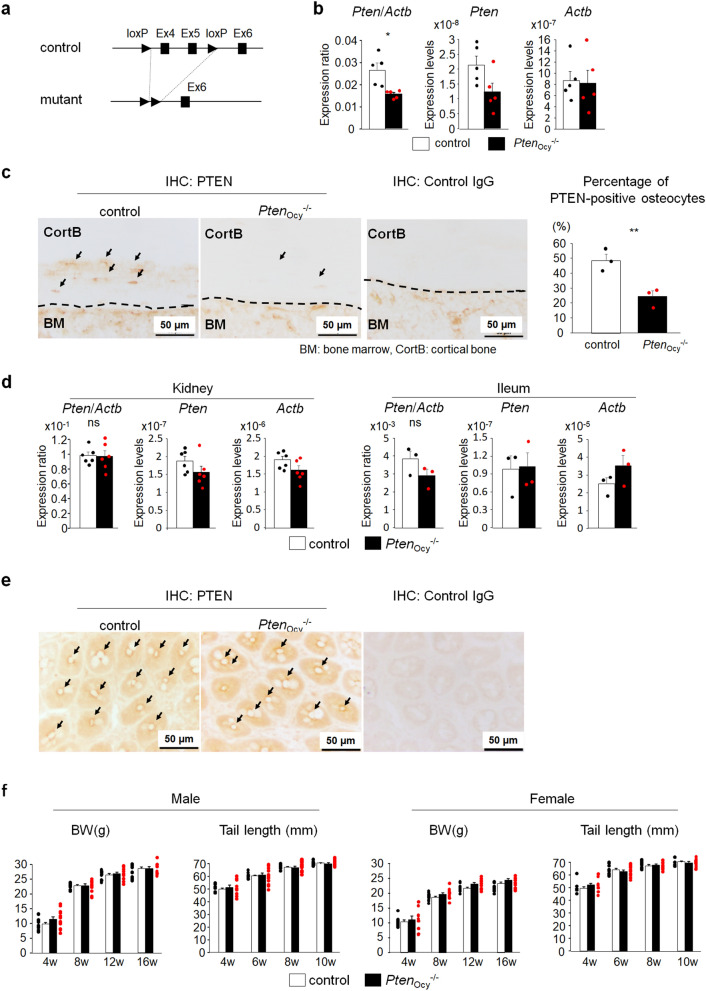
Generation of mice lacking PTEN expression in osteocytes. (**a**) Mice lacking *Pten* expression in osteocytes (*Pten*_Ocy_^−/−^ mice) were created by crossing *Pten*^flox/flox^ mice with *Dmp1*-Cre mice. (**b**) The expression of *Pten* and *Actb* in the whole femur was assessed by a real-time RT-PCR analysis at 16-week-oldmale mice (N = 5) and the expression ratio of *Pten*/*Actb* was determined. Statistical analysis was performed after corrected for the expression of *Actb*. (**c**) An immunohistochemical analysis of PTEN in the femur was performed on 16-week-old male mice and the percentage of PTEN-positive osteocytes over total osteocytes were determined. A representative image of three independent experiments is shown. Arrow indicates osteocytes positive for PTEN. (**d**) Total RNA was extracted from the kidney (N = 6) and the villi of the terminal ileum (N = 3) of 16-week-old male mice and the expression of *Pten* and *Actb* was investigated by a real-time RT-PCR analysis and the expression ratio of *Pten*/*Actb* was determined. Statistical analysis was performed after corrected for the expression of *Actb*. (**e**) An immunohistochemical analysis of PTEN in the terminal ileum was performed on 16-week-old male mice. A representative image of three independent experiments is shown. As indicated by arrows, basal surface of intestinal epithelial cells was positive for PTEN. (**f**) Body weight and tail length were measured at the indicated weeks in control and *Pten*_Ocy_^−/−^ mice from 4 weeks old (N = 11–13). Statistical analysis was performed by Mann–Whitney U test. *p < 0.01; **p < 0.05, ns: not significantly different.

To understand the role of PTEN in the regulation of Pi metabolism in osteocytes, we deleted *Pten* expression predominantly in osteocytes by crossing *Pten*^flox/flox^ mice with *Dmp1*-Cre mice (*Pten*_ocy_^−/−^ mice) (Fig. 1a). To evaluate the efficacy of the deletion in osteocytes, we initially examined the expression of *Pten* in the whole femur and found that it was significantly weaker in *Pten*_ocy_^−/−^ mice than in controls (*Pten*^flox/flox^ mice) (Fig. 1b). To specifically analyze the deletion of PTEN in osteocytes, we performed an immunohistochemical analysis and found that PTEN protein expression was decreased in the osteocytes of *Pten*_ocy_^−/−^ mice (Fig. 1c). We also investigated whether the use of *Dmp1* promoter-driven Cre-recombinase expression affected *Pten* expression in the extra-skeletal tissues involved in Pi metabolism, such as the kidney and terminal ileum. As shown in Fig. 1d, the expression of *Pten* in these tissues did not significantly differ between controls and *Pten*_ocy_^−/−^ mice. Since *Pten* expression in the terminal ileum showed a trend toward decreased expression, we performed immunohistochemical analysis of PTEN in the terminal ileum and found the comparable PTEN expression between control and *Pten*_ocy_^−/−^ mice (Fig. 1e). *Pten*_ocy_^−/−^ mice did not show any significant differences in body weight or tail length (Fig. 1f).

### The lack of PTEN in osteocytes decreases intact FGF23 expression in the bone and the circulation

**Figure 2:**
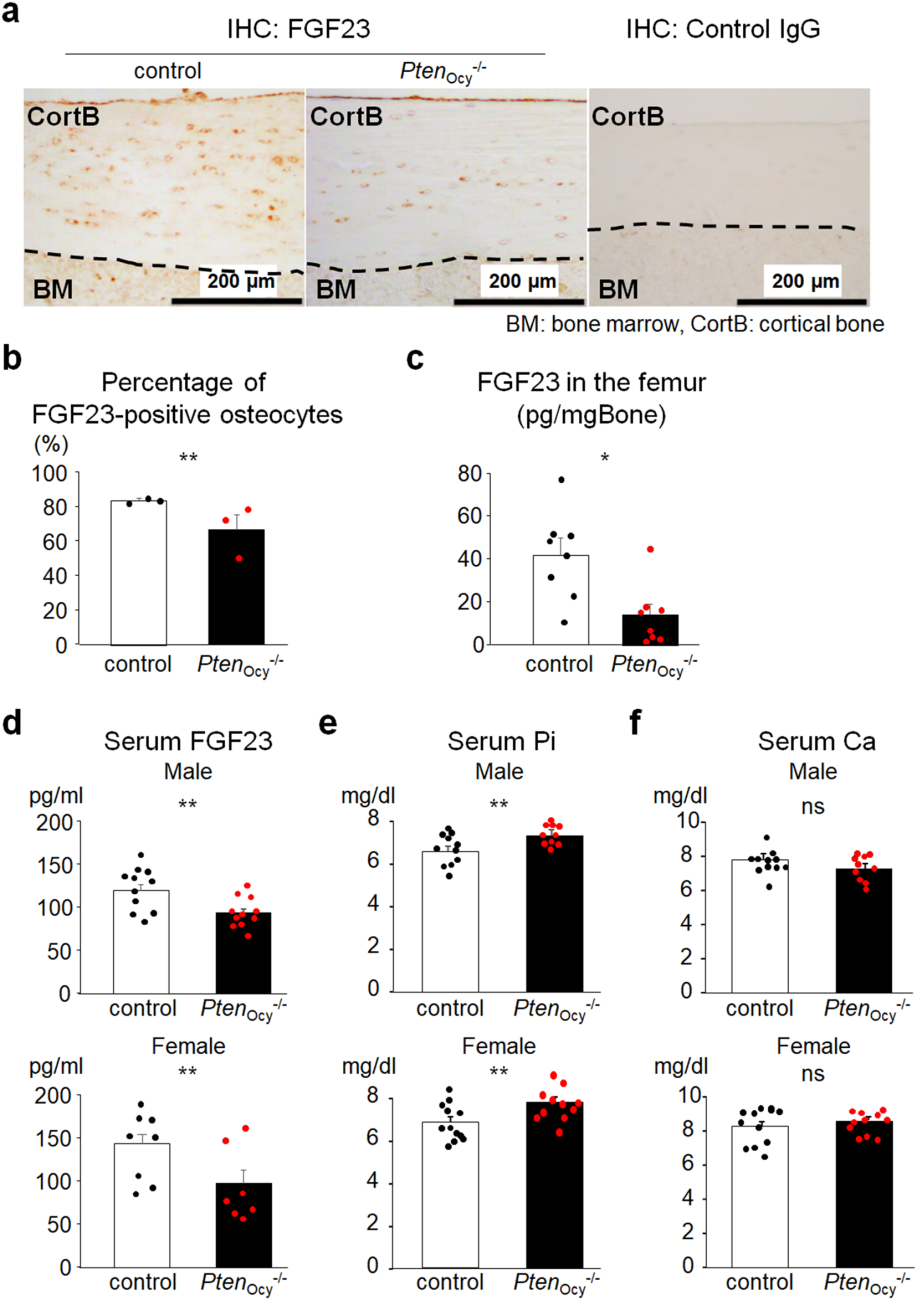
Decreased intact FGF23 levels in *Pten*_Ocy_^−/−^ mice. (**a**) and (**b**) An immunohistochemical analysis of FGF23 in the femur was performed on 16-week-old male mice. A representative image of three independent experiments is shown (**a**). The percentage of FGF23-positive osteocytes over total osteocytes was counted (N = 3) (**b**). (**c**) The bone extracts of the femur were prepared from 16-week-old male mice and intact FGF23 concentration was measured (N = 8). (**d–f**) Serum levels of intact FGF23 (male: N = 11, female: N = 7–8) (**d**), Pi (male: N = 10–11, female: N = 11–13) (**e**), and Ca (male: N = 10–11, female: N = 11–13) (**f**) were measured in 16-week-old mice. Statistical analysis was performed by Mann–Whitney U test. *p < 0.01; **p < 0.05, ns: not significantly different.

To investigate the effects of the *Pten* deletion in osteocytes in Pi metabolism, we examined FGF23 expression in the femur using immunohistochemistry and found that it was weaker in *Pten*_ocy_^−/−^ mice than in controls (Fig. 2a, b). In line with this, biologically active FGF23 (intact FGF23) concentration in the bone extracts was also decreased in *Pten*_ocy_^−/−^ mice compared to control mice (Fig. 2c). Intact FGF23 concentration in the serum was also decreased in *Pten*_ocy_^−/−^ mice (Fig. 2d). Consistent with decreased intact FGF23 levels in the circulation, serum Pi levels were elevated in *Pten*_ocy_^−/−^ mice (Fig. 2e). Serum Ca levels remained unchanged (Fig. 2f).

### The urinary excretion of Pi is elevated in *Pten*_ocy_^−/−^ mice

**Figure 3:**
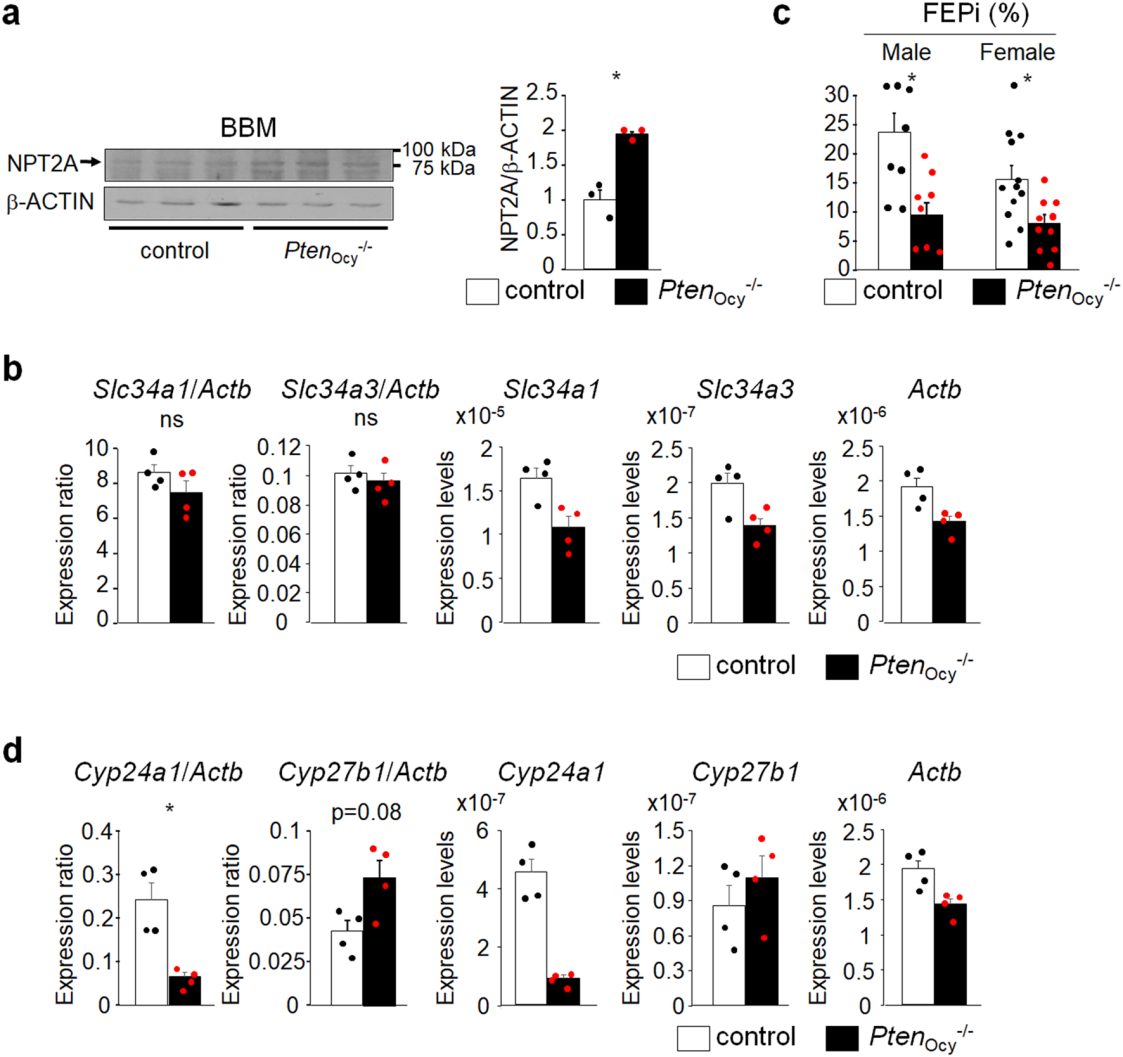
Enhanced Pi excretion in the urine of *Pten*_Ocy_^−/−^ mice. (**a**) The brush border membrane of the kidney was isolated from 16-week-old male mice and the expression of NPT2A was assessed by a Western blot analysis (N = 3). A densitometric analysis was performed (N = 3). (**b**) Total RNA was extracted from 16-week-old male mice and the expression of *Slc34a1*, *Slc34a3* and *Actb* in the kidney was assessed by real time RT-PCR (N = 4), and the expression ratio of *Slc34a1*/*Actb* and *Slc34a3*/*Actb* was determined. Statistical analysis was performed after corrected for the expression of *Actb*. (**c**) The fraction excretion of Pi was measured in 16-week-old mice (male: N = 8, female: N = 10–12). (**d**) Total RNA was extracted from 16-week-old male mice and the expression of *Cyp24a1*, *Cyp27b1* and *Actb* in the kidney was assessed by real time RT-PCR (N = 4), and the expression ratio of *Cyp24a1*/*Actb* and *Cyp27b1*/*Actb* was determined. Statistical analysis was performed after corrected for the expression of *Actb*. Statistical analysis was performed by Mann–Whitney U test. *p < 0.05, ns: not significantly different.

Since the nodal point of the regulation of Pi metabolism by the FGF23 signaling pathway resides in its effects on the subcellular localization of the type II Na^+^/Pi co-transporter (NPT2) protein, we evaluated the expression of NPT2A at the BBM of the kidney because NPT2A is a predominant Na^+^/Pi co-transporter in the murine kidney and found that it was increased in *Pten*_ocy_^−/−^ mice (Fig. 3a, Fig. S1); however, the expression of *Slc34a1* coding for NPT2A remained unchanged (Fig. 3b). The expression of *Slc34a3* in the kidney, coding for NPT2C, was much lower than that of *Slc34a1* and did not differ between control and *Pten*_ocy_^−/−^ mice (Fig. 3b). Consistent with increased NPT2A levels at the BBM of the kidney, the urinary excretion of Pi was significantly lower in *Pten*_ocy_^−/−^ mice than in controls (Fig. 3c). Since FGF23 also regulates vitamin D metabolism, we performed a real-time PCR analysis on the kidney and found that the expression of *Cyp24a1* and *Cyp27b1* was decreased and increased, respectively, in *Pten*_ocy_^−/−^ mice, although the difference in *Cyp27b1* expression did not reach statistical significance (Fig. 3d).

### Knockdown of PTEN expression in UMR106 cells reduces *Fgf23* expression

**Figure 4:**
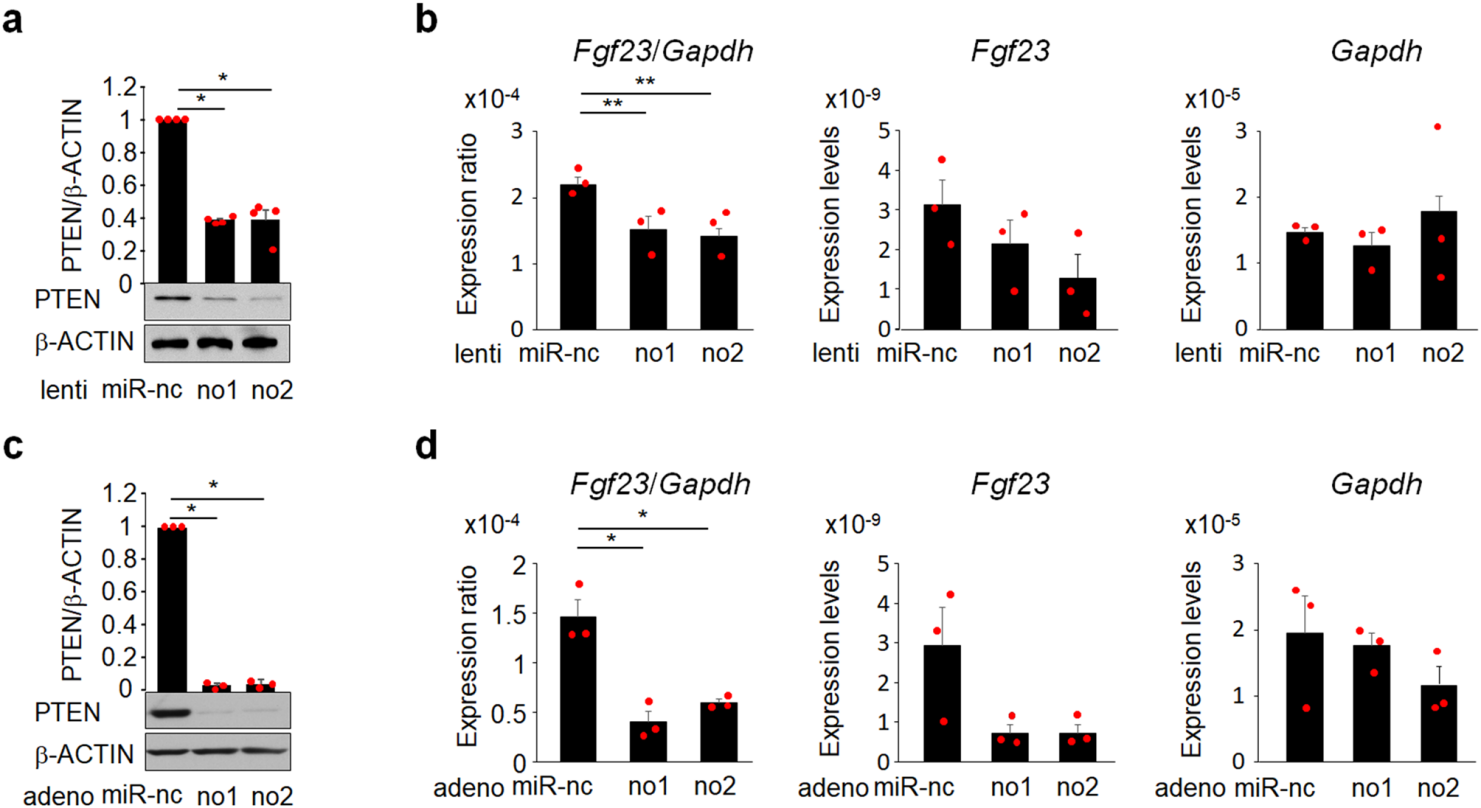
The lack of PTEN in UMR106 cells suppresses *Fgf23* expression. (**a**) and (**b**) The expression of PTEN was knocked down using a lentivirus and knockdown efficacy was evaluated by a Western blot analysis for PTEN (N = 4) (**a**). The expression of *Fgf23* and *Gapdh* was assessed by a real-time RT-PCR analysis and the expression ratio of *Fgf23*/*Gapdh* was determined. Statistical analysis was performed after corrected for the expression of *Gapdh*. (N = 3) (**b**). (**c**) and (**d**). The expression of PTEN was knocked down using an adenovirus and knockdown efficacy was evaluated by a Western blot analysis for PTEN (N = 3) (**c**). The expression of *Fgf23* and *Gapdh* was assessed by a real-time RT-PCR analysis and the expression ratio of *Fgf23*/*Gapdh* was determined. Statistical analysis was performed after corrected for the expression of *Gapdh*. (N = 3) (**d**). Statistical analysis was performed using one-way ANOVA. *p < 0.01; **p < 0.05, miR: microRNA, nc: negative control.

In order to elucidate the molecular mechanisms underlying FGF23 regulation by PTEN, we knocked down PTEN expression in osteoblastic UMR106 cells. The lentiviral introduction of miRNA raised against *Pten* induced a mild reduction in PTEN expression (Fig. 4a, Fig. S2), which was associated with mild reduction in *Fgf23* expression (Fig. 4b). When PTEN expression was more strongly knocked down in UMR106 cells using an adenovirus (Fig. 4c, Fig. S2), the suppression of PTEN expression was more prominent (Fig. 4d). These results indicate that the lack of PTEN in osteoblastic cells reduces *Fgf23* expression in a dose-dependent manner. Since the reduction of PTEN expression by lentivirus was similar to that observed in bones of *Pten*_ocy_^−/−^ mice, UMR106 cells in which PTEN expression was knocked-down by lentivirus were used in the subsequent experiments.

### Rapamycin partially restores decreased *Fgf23* expression caused by PTEN knockdown or insulin stimulation in UMR106 cells

**Figure 5:**
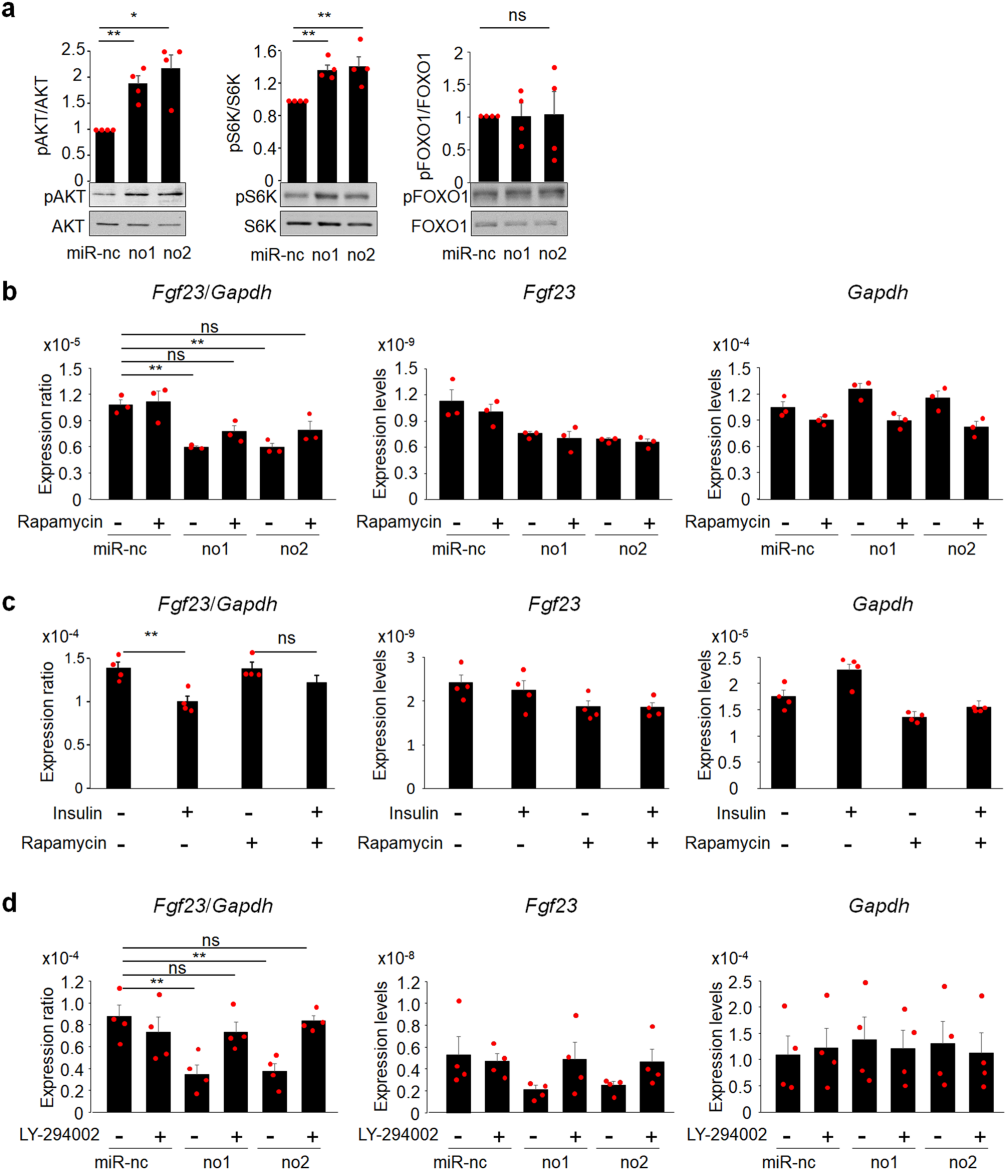
Suppression of *Fgf23* expression by PTEN knockdown or insulin was partially restored in the presence of rapamycin in UMR106 cells. (**a**) The expression of PTEN was knocked down using a lentiviral system in UMR106 cells and the expression of pAKT/AKT, pS6K/S6K, and pFOXO1/FOXO1 was assessed by a Western blot analysis, which was followed by a densitometric analysis (N = 4). (**b**) The expression of PTEN was knocked down using a lentiviral system in UMR106 cells and treated with 10 nM of rapamycin (**b**). The expression of *Fgf23* and *Gapdh* was assessed by a real-time RT-PCR analysis and the expression ratio of *Fgf23*/*Gapdh* was determined. Statistical analysis was performed after corrected for the expression of *Gapdh* (N = 3). (**c**) UMR106 cells were pre-treated with 10 nM of rapamycin for 6 h, followed by an overnight treatment with 50 nM of insulin. The expression ratio of *Fgf23*/*Gapdh* was determined. Statistical analysis was performed after corrected for the expression of *Gapdh* (N = 4). (**d**) The expression of PTEN was knocked down using a lentiviral system in UMR106 cells and treated with 5 μM of LY-294002. The expression of *Fgf23* and *Gapdh* was assessed by a real-time RT-PCR analysis and the expression ratio of *Fgf23*/*Gapdh* was determined. Statistical analysis was performed after corrected for the expression of *Gapdh* (N = 4). Statistical analysis was performed using one-way ANOVA. *p < 0.01; **p < 0.05, ns: not significantly different.

To identify the downstream signaling molecules involved in *Fgf23* suppression due to the lack of PTEN, we performed a Western blot analysis and found that the phosphorylation status of AKT and S6K was elevated in cells in which PTEN was knocked down, suggesting that the AKT/mTORC1 pathway was more strongly activated in PTEN knockdown cells than in control cells, whereas the phosphorylation of FOXO1 was not affected in the condition where PTEN was mildly knocked down (Fig. 5a, Fig.S3). To investigate the involvement of the activation of mTORC1, we treated cells with rapamycin, an mTORC1 inhibitor, and found that the significant reduction in *Fgf23* expression by PTEN knockdown was not noted in the presence of rapamycin, but rapamycin did not fully restore decreased *Fgf23* expression (Fig. 5b). Consistent with this, insulin, an activator of the AKT/mTORC1 pathway, reduced *Fgf23* expression, but this was not observed in the presence of rapamycin (Fig. 5c). The use of LY-294002, a Pi3K inhibitor, restored the reduction in *Fgf23* expression caused by PTEN knockdown more prominently than rapamycin (Fig. 5d). These findings indicate that the activation of the mTORC1 pathway is at least partly involved in the regulation of *Fgf23* expression although other pathways than mTORC1 also play an important role in this regulation.

## Discussion

In the present study, we tested our hypothesis on the involvement of AKT activation in the regulation of Pi metabolism by deleting PTEN expression in an osteocyte-specific manner and provided evidence to show that the lack of PTEN in osteocytes caused elevations in serum Pi levels by reducing circulating FGF23 concentrations. In addition, an in vitro analysis revealed the nodal point of mTORC1 activation in this regulation, such that the treatment with rapamycin, an inhibitor of the mTORC1, resulted in the partial rescue of *Fgf23* suppression by PTEN knockdown or insulin stimulation in UMR106 cells. These lines of evidence support the activation of the AKT/mTORC1 pathway in osteoblastic cells being involved in the regulation of FGF23 expression.

These results are partly consistent with previous findings, in which the activation of the AKT signaling pathway by insulin or IGF-1 reduced *Fgf23* expression in UMR106 cells through the FOXO1 signaling pathway^17^. In vivo insulin depletion by streptozotocin in mice increased C-terminal FGF23, which includes the biologically active and inactive forms of FGF23; however, serum Pi levels were unaffected^17^, which raises questions regarding the functional interaction between osteocytic AKT activation and Pi metabolism. In contrast, we herein provided in vivo evidence to show that the activation of the AKT pathway through the suppression of PTEN has functional consequences in Pi metabolism; the lack of PTEN in osteocytes decreases biologically active intact FGF23, which, in turn, increases serum Pi levels. In addition, increases in the BBM localization of NPT2A and decreases in *Cyp24a1* expression were functionally consistent with decreased serum intact FGF23 levels. These results together with previous findings support the critical role of the AKT signaling pathway in osteocytes in the regulation of Pi metabolism through alterations in FGF23 expression.

The results of the in vitro analysis showed that mTORC1 activation was involved in the insulin-induced suppression of *Fgf23* expression in UMR106 cells; however, the in vivo relevance of mTORC1 activity in the regulation of FGF23 expression is limited. Based on clinical evidence obtained from patients after kidney transplantation, the use of rapamycin, an mTORC1 inhibitor, has been shown to cause hypophosphatemia associated with the impaired renal reabsorption of Pi^20,21^; however, this has not been associated with alterations in FGF23 levels^20^. To understand the effects of mTORC1 activation on Pi metabolism, the in vivo administration of an mTORC1 inhibitor in animal models has been performed^22,23^. Haller et al. reported that a treatment with sirolimus, an mTORC1 inhibitor, in rats resulted in decreases in serum Pi levels and increases in urinary Pi excretion^22^. Interestingly, FGF23 levels were unchanged 2 days after the sirolimus treatment, but were reduced on day 7, suggesting that decreases in FGF23 were secondary to low Pi levels. As described above, previous studies have consistently demonstrated the phosphaturic effects of systemic mTORC1 inhibition; however, the underlying mechanisms remain largely unknown, although NPT2A/2C localization was shown to be unaffected by mTORC1 inhibition^22,23^. These findings indicate that the systematic introduction of an mTORC1 inhibitor may have stronger effects in proximal tubules than in osteocytes and, thus, the direct effects of mTORC1 inhibition on FGF23 production in osteocytes become minimized. Additional studies, such as the deletion of the mTORC1 component in osteocytes, will clarify the role of mTORC1 activation in the regulation of FGF23 in osteocytes.

In the present study, we did not find any difference in the phosphorylation status of FOXO1 between control and PTEN-knocked down UMR106 cells, and this may in part be due to the mild knockdown efficacy of PTEN. We took advantage of this cell model to delineate the signaling cascades other than FOXO1 signaling pathway that may regulate *FGF23* expression and found the involvement of mTORC1 in the regulation of *Fgf23* expression in UMR106 cells. However, this result does not necessarily mean that signaling pathway other than mTORC1 is not involved in this regulation. Indeed, the treatment with rapamycin did not fully restore the suppression of *Fgf23* by PTEN knockdown or the insulin stimulation, whereas the treatment with a Pi3K inhibitor showed greater increase in *Fgf23* expression in PTEN-knocked down UMR106 cells. In addition, there is a conflicting paper describing that rapamycin reduced *Fgf23* expression in UMR106 cells treated in high-glucose (4.5 g/dL) media^24^, which was not consistent with the present result describing that rapamycin did not show any alteration in *Fgf23* expression in control UMR106 cells cultured in high-glucose media. These differences may represent the disadvantage of the use of UMR106 cells because UMR106 cells express very low level of *Fgf23*; therefore, other models including IDG-SW3 cells may be necessary to draw concrete conclusion regarding the role for mTORC1 pathway in the regulation of *FGF23*.

The clinical relationship between FGF23 and metabolic markers has been emerging. For example, a positive relationship has been implied between FGF23 levels and BMI or adiposity^25,26^, and leptin, PTH, vitamin D, and inflammatory signaling have been suggested to play a role in this regulation. Leptin levels were previously shown to be elevated in obese subjects and positively correlated with FGF23^27^. Furthermore, leptin has been implicated as a positive regulator of FGF23 levels in humans^28^. A relationship was also noted between FGF23 and 1,25(OH)_2_D levels^27^, which is known to transcriptionally increase *FGF23* expression. These findings indicate a connecting role for leptin and/or 1,25(OH)_2_D between obesity and FGF23. PTH may also be a candidate factor for this connection because PTH levels are elevated in obese subjects^28,29^ and there is evidence to show that PTH is a positive regulator for FGF23^12,13^. However, Billington et al. recently reported that FGF23 concentrations decreased after sleeve gastrectomy independent of changes in adiposity, PTH, and leptin levels^30^, which suggests an additional factor affecting FGF23 concentrations in humans. Based on accumulating evidence showing a relationship between insulin and FGF23 levels in humans^17,18^, insulin signaling has been implicated in the regulation of FGF23. A recent study on healthy volunteers demonstrated that the area under the curve of FGF23 levels was inversely associated with that of insulin after an oral glucose tolerance test^17^. Similarly, a negative correlation was observed between FGF23 and both C-peptide and fasting insulin in obese subjects^31^.

The factors that activate AKT signaling and decrease FGF23 expression in osteocytes were not identified in the present study. As described above, insulin is a potential candidate regulating AKT signaling in osteocytes. In support of this notion, the presence of insulin receptors in UMR106-01 cells, not in immature osteoblastic UMR201-10B cells, was previously reported^32^. Similarly, insulin receptor expression was detected in the osteocyte-like cell line IDG-SW3^33^. Despite these findings, in vivo evidence for the expression of insulin receptors in osteocytes is limited. Although there are several models of insulin receptor deletion in osteoblastic cells^34–36^, FGF23 and Pi levels have not yet been measured in these mice. Insulin-like growth factor 1(IGF-1) is also a potential factor for this regulation. Interestingly, the deletion of *Igf1* in osteocytes resulted in higher FGF23 levels than those in control mice^37^, suggesting a role for IGF-1 in this regulation. Further studies are needed to clarify the involvement of insulin or IGF-1 in the regulation of FGF23 expression in osteocytes.

In summary, we herein provided evidence for the critical role of PTEN suppression, mainly in osteocytes, in the regulation of Pi metabolism through the modulation of FGF23 levels, which may be attributed to the activation of the AKT/mTORC1 signaling pathway; however, the clinical implications of this regulation remain largely unknown. Due to accumulating evidence for the relationship between disorders associated with aberrant insulin signaling and FGF23 levels, the present results may provide novel insights into the role of osteocytic AKT activation in the regulation of FGF23 expression, which may be critical for the regulation of Pi homeostasis.

## Methods

### Ethical considerations

All animal studies were reviewed and approved by the Institutional Animal Care and Use Committee of the Osaka Women’s and Children’s Hospital (Permit No. BMR-2018-1), and all experiments were performed in accordance with relevant guidelines and regulations.

### Animal

*Pten*^flox/flox^ mice were generated as previously described^38^, and kindly provided by Dr. Tak W Mak (Princess Margaret Cancer Centre, University Health Network, Toronto, Canada). The generation of 10-kb *Dmp1*-Cre mice was previously described^39^. To generate osteocyte-specific *Pten* knockout mice (*Pten*_ocy_^−/−^ mice), *Pten*^flox/flox^ mice on a C57BL/6J background were crossed with *Dmp1*-Cre mice on a C57BL/6J background. *Pten*^flox/flox^ mice were used as control mice for *Pten*_ocy_^−/−^ mice. Mice were maintained with free access to water and standard chow containing 1.0% phosphate and 1.0% calcium (CE-2, CLEA Japan, Inc.) on a 12-h:12-h light/dark cycle in a pathogen-free animal facility.

### Cell culture

UMR-106 cells were purchased from ATCC (Manassas, VA) and cultured in high-glucose DMEM containing 10% fetal bovine serum and 1% insulin-transferrin-selenium-G supplement (Invitrogen, Carlsbad, CA).

### Western blot analysis

To prepare whole cell lysates, UMR106 cells were homogenized in RIPA buffer (1% Triton, 1% Na deoxycholate, 0.1% SDS, 150 mM NaCl, 10 mM Tris–HCl (pH 7.4), 5 mM EDTA, 1 mM orthovanadate, and protease inhibitor cocktail (cOmplete, Mini, EDTA-free Protease Inhibitor Cocktail, Roche) and centrifuged. The supernatant was used as the whole cell lysate. The BBM of the kidney was isolated based on the divalent cation precipitation method as previously reported^40^. In brief, the kidney was homogenized in 30 volumes (w/v) of homogenization buffer (50 mM mannitol and 2 mM Tris–HCl, pH 7.5), followed by the incubation of homogenates with 100 mM CaCl_2_ with rotation at 4 °C for 10 min and centrifugation at 3,000×*g* at 4 °C for 15 min. The supernatant was collected and again centrifuged at 43,000×*g* at 4 °C for 30 min. The pellet was resuspended in suspension buffer (300 mM mannitol and 10 mM Tris-HEPES, pH 7.5) and homogenized, followed by centrifugation at 43,000×*g* at 4 °C for 45 min. The pellet was solubilized in RIPA buffer and used as the BBM fraction.

Equal amounts of protein were separated by SDS-PAGE and transferred electrophoretically to PVDF membranes. Blocking was performed using BlockAce reagent (Dainippon Pharmaceuticals, Osaka, Japan) or Blocking-one P reagent (Nacalai Tesque). Membranes after SDS-PAGE were then immunoblotted with antibodies raised against AKT (1:1000, #9272, Cell Signaling), pSer473-AKT (1:1000, #9271, Cell Signaling), S6K (1:1000, #2708, Cell Signaling), pS6K (1:1000, #9205, Cell Signaling), FOXO1 (1:1000, #2880, Cell Signaling), pSer256-FOXO1(1:1000, #9461, Cell Signaling), PTEN (1:1000, #9559, Cell Signaling), GAPDH (1:2000, sc-20357, Santa Cruz Biotechnology), or β-ACTIN (1:2000, sc-47778, Santa Cruz Biotechnology), and developed with horseradish peroxidase-coupled secondary antibodies, followed by enhancements with a chemiluminescence (ECL) detection system (GE Healthcare). An anti-NPT2A antibody (rabbit polyclonal antibody) was raised against an NPT2A peptide sequence (MMSYSERLGGPAVSP) in the amino-terminal region^41^.

### Real-time RT-PCR

Total RNA was prepared using TRIzol (Invitrogen) and treated with DNase I (Qiagen). cDNA was generated using a random hexamer and reverse transcriptase (Superscript II, Invitrogen) according to the manufacturer’s instructions. The quantification of mRNA expression was performed using a StepOnePlus Real-time PCR system (Applied Biosystems). TaqMan Gene Expression Assays for mouse *Slc34a1*, rat *Fgf23*, mouse *Gapdh*, mouse *Actinb*, and rat *Gapdh* were purchased from Applied Biosystems. Primer sequences for mouse *Pten* are available upon request. *Actinb* or *Gapdh* was used as an internal standard control gene for quantifications. Expression level of each gene was evaluated by generating a standard curve with the use of serial dilution of plasmid DNA containing the PCR product of interest (10^–10^, 10^–9^, 10^–8^, 10^–7^ and 10^–6^ μg/μL).

### Generation of the lentivirus and adenovirus

The generation of the lentivirus and adenovirus was performed as previously reported^40^. Knockdown experiments for *Pten* were performed based on the lentivirus-mediated expression of the microRNA (miRNA) system using the BLOCK-iT Pol II miR RNAi Expression Vector Kit with EmGFP (Invitrogen). The sequences of DNA oligos for this purpose were designed using BLOCK-iT RNAi Designer provided by the manufacturer and hybridized oligos were inserted in the pcDNA6.2-GW/EmGFP-miR vector, followed by transfer into the pDONR vector by the BP reaction system (Invitrogen). To generate the lentivirus, the insert was recombined into the CSII-EF-RfA–IRES-puro vector (a kind gift from Dr. Hiroyuki Miyoshi (RIKEN BRC, Ibaraki, Japan) with the LR recombination reaction system (Invitrogen). The lentivirus was generated according to the manual provided by RIKEN BRC. UMR106 cells were infected with the lentivirus in the presence of 5 μg/mL polybrene (Sigma) and selected by puromycin (1 μg/mL). In the case of the generation of the adenovirus, the insert was recombined into the pAd-CMV-V5 vector (Invitrogen) using the LR recombination reaction system (Invitrogen). The adenovirus was generated using the ViraPower Adenovirus Expression System (Invitrogen) according to the manufacturer’s protocol.

### Immunohistochemistry

Tissue samples were fixed in 10% buffered formalin and decalcified by the treatment with EDTA (pH7.4). Paraffin-embedded samples were prepared. In the immunohistochemical analysis, following deparaffinization and rehydration, antigen retrieval was performed using citrate buffer at 95 °C for 60 min and endogenous peroxidase activity was quenched. In case of FGF23 immunohistochemistry, antigen retrieval was not performed. After blocking, sections were incubated with an anti-PTEN antibody (1:16, #9559, Cell Signaling) or anti-FGF23 antibody (1:20, #MAB26291, R&D systems) at 4 °C overnight. Sections were then incubated with a biotinylated secondary antibody, followed by an incubation with the streptavidin-biotinylated HRP complex, and visualized with 3, 3′-diaminobenzidine.

### Extraction of growth factors from the femur and measurements of FGF23 concentration

Femurs were collected from 16-week-old male mice and bone marrow was removed by rinsing with ice-cold PBS. Bones were snap-frozen in liquid nitrogen and ground into powders using a cryo-press. The bone powder was transferred to siliconized tubes in the presence of 2.0 mL of extraction solution (0.5M EDTA, 4M guanidine HCl, and protease inhibitor cocktail (cOmplete, Mini, EDTA-free Protease Inhibitor Cocktail, Roche) per 50 mg bone powder and rotated at 4 °C for 48 h. The supernatant was collected after centrifuge and dialyzed against phosphate-buffered saline (PBS) for 48 h at 4 °C, and intact FGF23 was measured using an ELISA kit (KAINOS Laboratories, Inc.).

### Measurement of serum parameters

Serum levels of full-length FGF23 were measured using the intact FGF-23 ELISA Kit based on the manufacturer’s protocol (KAINOS Laboratories, Inc.). Serum and urinary phosphate, calcium, and creatinine levels were measured by Phospha-C test Wako (270-49801, Wako Pure. Chemical Industries), Calcium-E test Wako (437-58201, Wako Pure. Chemical Industries), and LabAssay Creatinine (290-65901, Wako Pure. Chemical Industries), respectively, following the manufacturers’ instructions.

### Statistical analysis

All data are expressed as the mean ± standard error of the mean (SEM). Results were analyzed for significant differences using Mann–Whitney U test or ANOVA followed by the Bonferroni multiple comparison post hoc test. Significance was set at *p* < 0.05.

## Data availability

The datasets generated during and/or analyzed during the current study are available from the corresponding author on reasonable request.

## Supplementary information

Supplementary Figures.
