# Supplementary material for: Lack of PTEN in osteocytes increases circulating phosphate concentrations by decreasing intact fibroblast growth factor 23 levels
Source: Sci Rep. 2020 Dec 9;10:21501. doi: 10.1038/s41598-020-78692-6 (PMC7726559; doi:10.1038/s41598-020-78692-6)
Supplement: Supplementary file 1 — Supplementary Figures. [file 41598_2020_78692_MOESM1_ESM.pdf]

Supplementary material

Lack of PTEN in osteocytes increases circulating phosphate concentrations by decreasing intact fibroblast growth factor 23 levels

Masanobu Kawai[1, *], Saori Kinoshita[1, *], Keiichi Ozono[2], and Toshimi Michigami[1]

1. Department of Bone and Mineral Research, Research Institute, Osaka Women's and Children's Hospital, Osaka, Japan

2. Department of Pediatrics, Osaka University Graduate School of Medicine

*. These authors contributed equally to this work.

Corresponding author

Masanobu Kawai, MD, PhD

Department of Bone and Mineral Research, Research Institute, Osaka Women's and Children's Hospital, 840 Murodo-cho, Izumi, Osaka 594-1101, Japan

Tel.: +81-725-56-1220

Fax: +81-725-57-3021

E-mail: kawaim@wch.opho.jp

Supplementary Figure S1

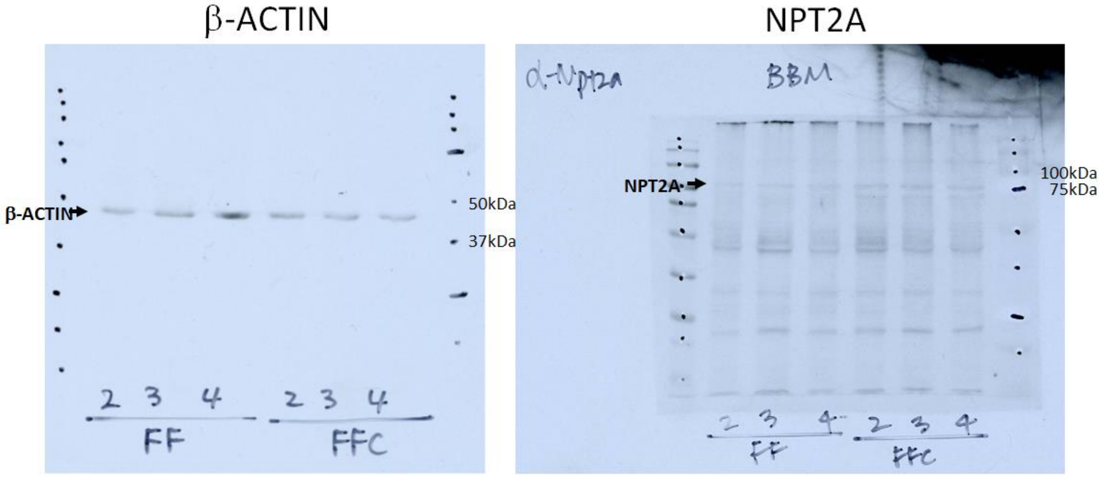

FF: control *FFC: Pten*$_{Ocy}^{-/-}$

Supplementary Figure S1. Full-size gels for Fig. 3a

Supplementary Figure S2

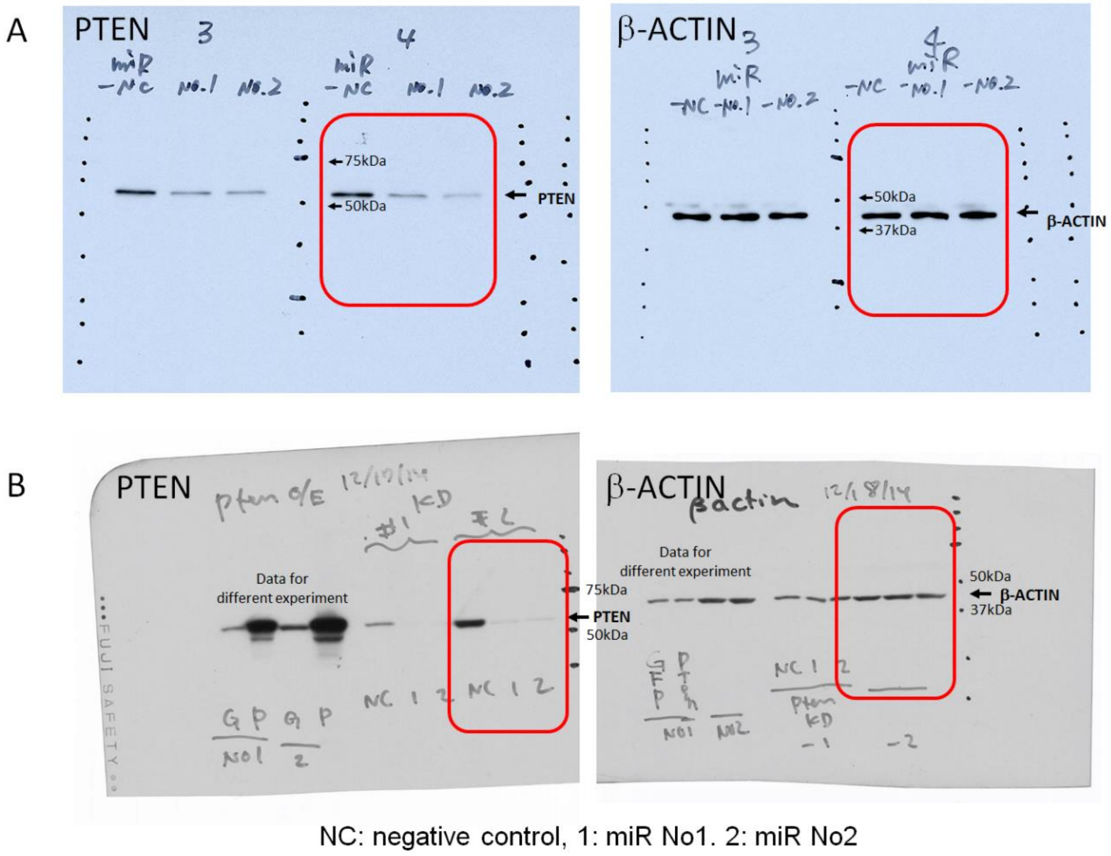

Supplementary Figure S2. Full-size gels for Figs. 4a and 4c

Blots highlighted by red-colored square are used in the Figs. 4a and 4c.

Supplementary Figure S3

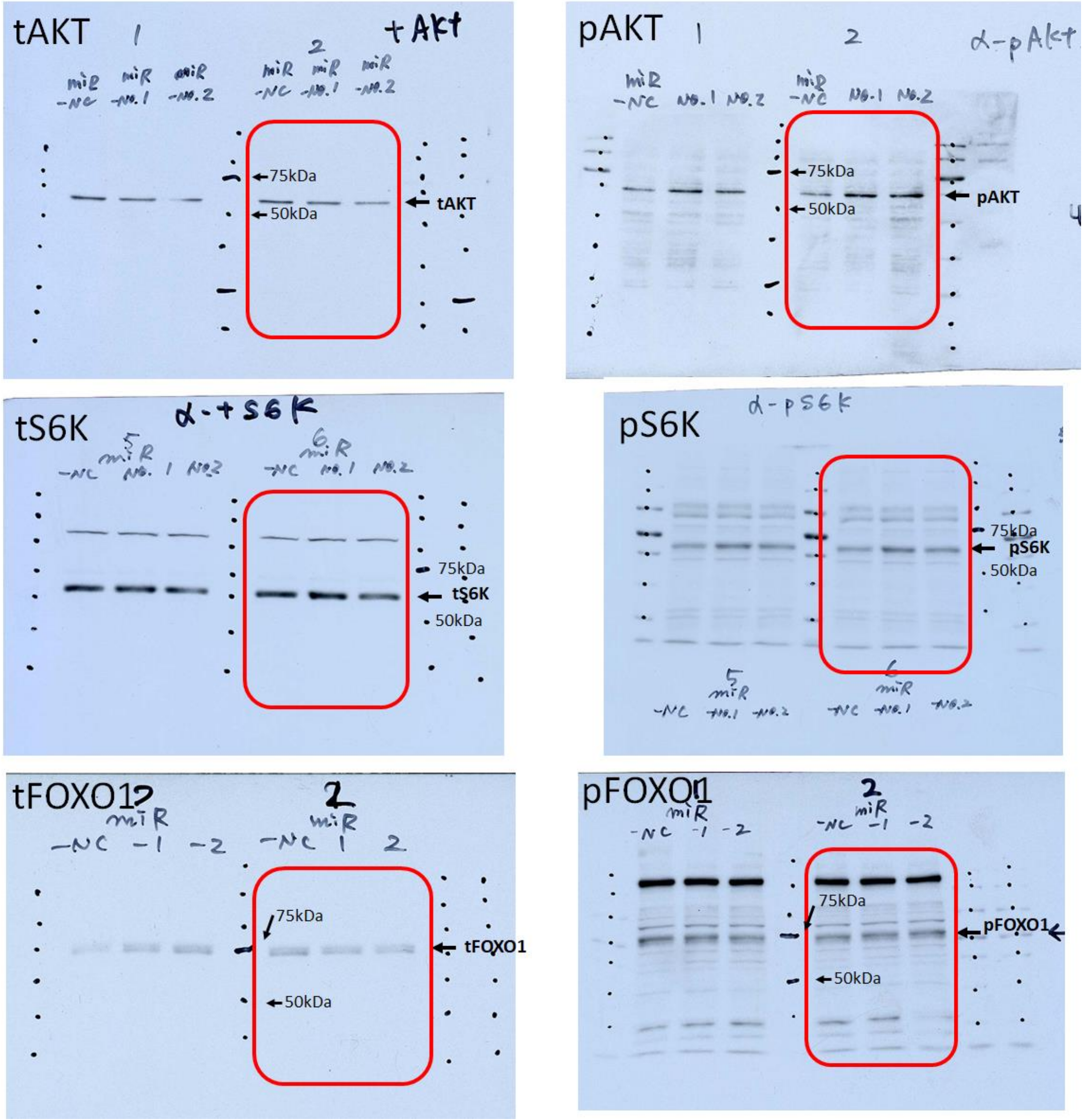

Supplementary Figure S3. Full-size gels for Fig. 5a.

Blots highlighted by red-colored square are used in the Fig. 5a.
